# Fabrication of copper(II)-coated magnetic core-shell nanoparticles Fe_3_O_4_@SiO_2_-2-aminobenzohydrazide and investigation of its catalytic application in the synthesis of 1,2,3-triazole compounds

**DOI:** 10.1038/s41598-021-81632-7

**Published:** 2021-01-22

**Authors:** H. Rajabi-Moghaddam, M. R. Naimi-Jamal, M. Tajbakhsh

**Affiliations:** Research Laboratory of Green Organic Synthesis and Polymers, Department of Chemistry, Iran University of Science and Technology, P.O. Box 16846–13114, Tehran, Iran

**Keywords:** Catalysis, Organic chemistry

## Abstract

In the present work, an attempt has been made to synthesize the 1,2,3-triazole derivatives resulting from the click reaction, in a mild and green environment using the new copper(II)-coated magnetic core–shell nanoparticles Fe_3_O_4_@SiO_2_ modified by isatoic anhydride. The structure of the catalyst has been determined by XRD, FE-SEM, TGA, VSM, EDS, and FT-IR analyzes. The high efficiency and the ability to be recovered and reused for at least up to 6 consecutive runs are some superior properties of the catalyst.

## Introduction

**Figure 1:**
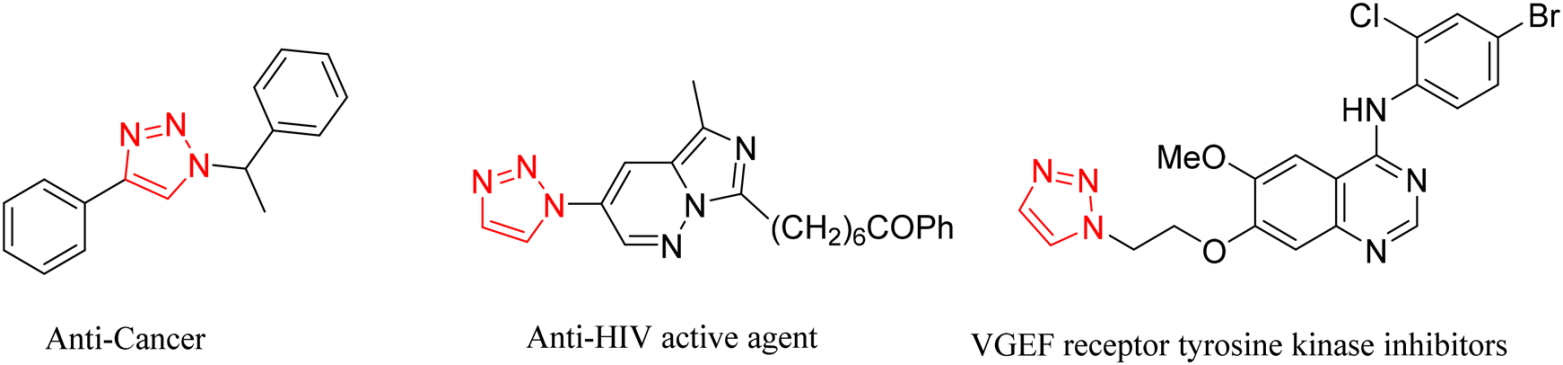
Bioactive molecules with 1,2,3-triazole ring.

Nitrogen-containing heterocyclic combinations, especially five-membered 1,2,3-triazole structures, are present in a variety of compounds including pigments, biologically active compounds (e.g., inhibitors and anti-cancers), agrochemicals, explosives, and antibacterial and antifungal drugs^1–9^. Figure 1 shows some compounds containing this ring. A. Michael first reported the synthesis of such compounds in 1893^10^.

**Figure 2:**
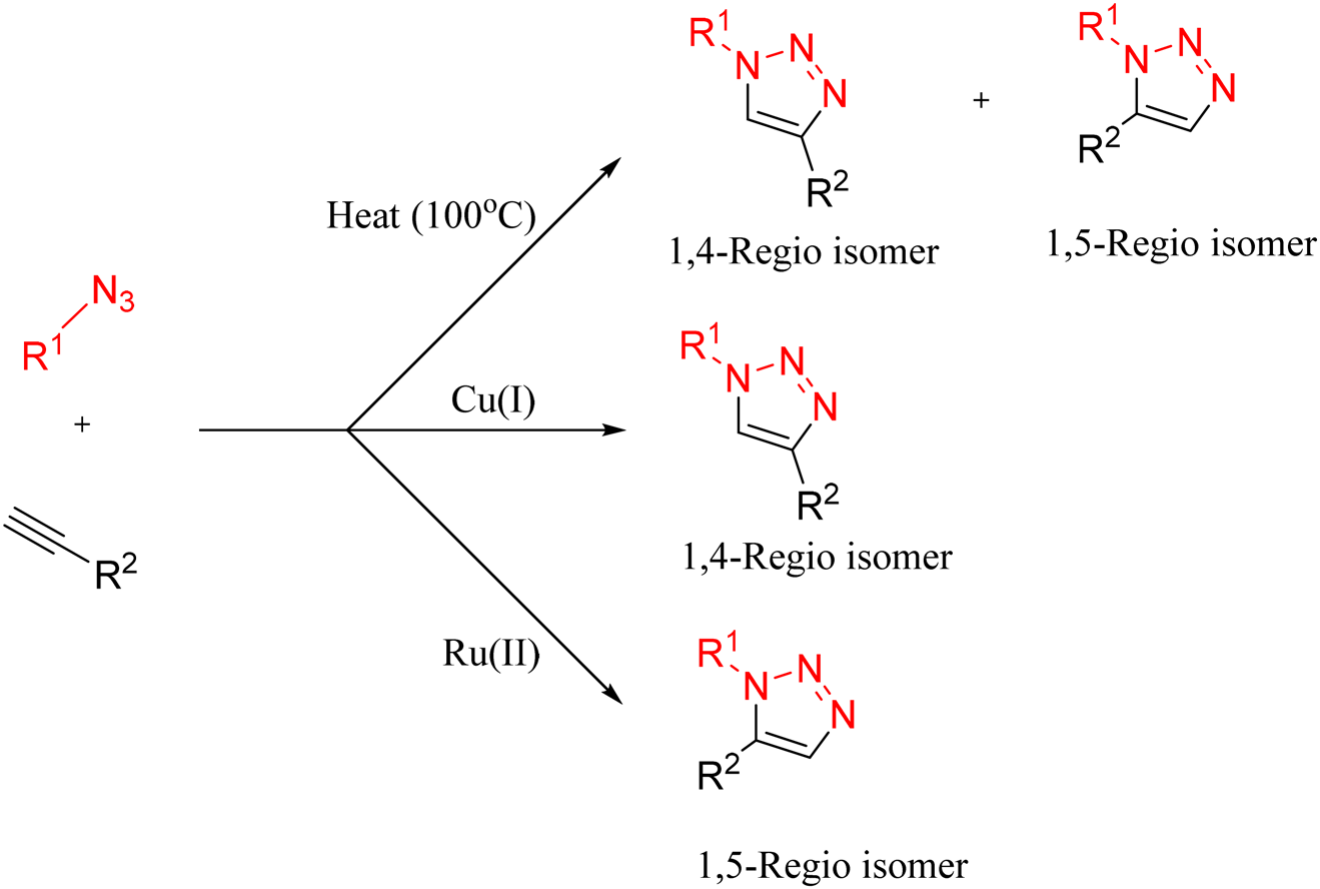
Ruthenium and copper-catalyzed azide-alkyne cycloaddition.

1,3-Dipolar Huisgen cycloaddition reaction of azides with alkynes was investigated in 1960 with Cu(I) and ruthenium(II) (known as Cu-AAC and Ru-AAC reactions). This reaction is shown in Fig. 2^11^. By this reaction, 1,4- or 1,5-disubstituted 1,2,3-triazoles are prepared with high regiospecifity. Then Sharpless discovered Cu(I)-catalyzed cycloaddition in 2002^12^. This method is the most general and popular method among others^13,14^. In 2010, zinc was also used efficiently as a catalyst in this type of reaction, but the yield of products was low^15^. In recent years, due to the importance of these compounds, several methods for making them quickly and easily have been proposed by organic chemists. *Nandi et. al.* in 2016 studied the light effect on a click reaction, which is a way to accelerate product formation and is environmentally safe^16^.

Due to the complexity of using Cu(I), e.g. disproportionation reactions, need to the inert atmosphere, formation of undesired by-products, etc*.* development of new methods for producing Cu(I) catalyst in situ by the reduction of Cu(II) or oxidation of Cu(0) to Cu(I) was expanded^17^.

Recently, the use of magnetic heterogeneous metal catalysts in organic reactions has been developed. There are several reasons for this, including high stability, reusability, and ease of separation from the reaction media. It should be noted that the efficiency of homogeneous catalysts is higher than that of heterogeneous catalysts due to the proper treatment of reactants. But the absence of recyclability and difficult separation from the reaction mixture has led scientists to use heterogeneous catalysts. For this purpose, solid-supported catalysts with different bases like (bio)polymers^18–20^, clays and zeolites^21,22^, etc. have been reported to perform this reaction. *Esmaeilpour, et. al.* in 2016 used 1,4-dihydroxyanthraquinone–copper(II) supported on superparamagnetic Fe_3_O_4_@SiO_2_ in this reaction^23^. This reaction was also performed by the *Ranu* group in the solvent-free condition under ball-milling over a Cu/Al_2_O_3_ surface as a heterogeneous catalyst^24^.

In this work, we report on a new silica-supported magnetic copper catalyst used in 1,2,3-triazole synthesis. How to prepare, the identification and characterization and the application in AAC reaction are described below.

## Results and discussions

**Scheme 1:**
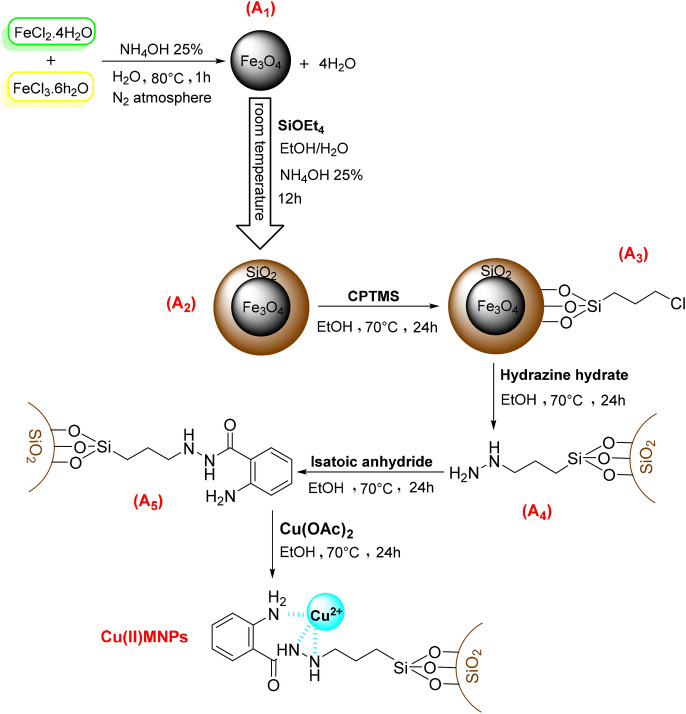
Catalyst synthesis steps.

Fe_3_O_4_ nanoparticles were synthesized using the co-precipitation method reported in the literature^25,26^. In the next step, to prepare the core–shell nanoparticles, silica coating was done under the Stober process. Then, the core–shell nanoparticles have been modified by isatoic anhydride (Scheme 1).

**Figure 3:**
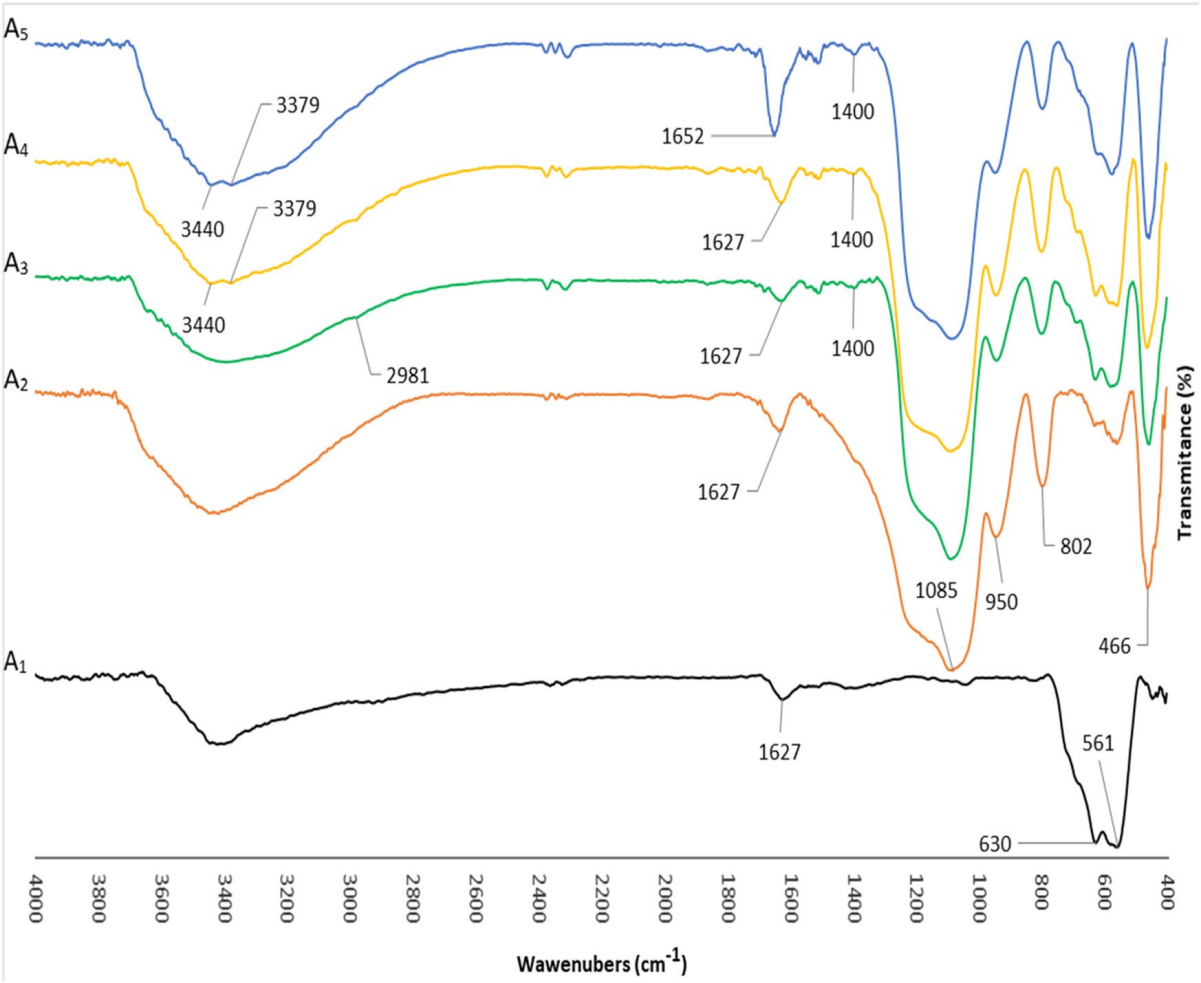
Comparative study of FT-IR spectra for (A_1_) to (A_5_).

The FT-IR spectra corresponding to each stage of catalyst preparation are shown in Fig. 3. In the spectrum of sample A_1_, the two bands exhibited at 561, and 630 cm^−1^ are attributed to the Fe–O stretching vibration. Furthermore, the band located at 1627 cm^−1^ is assigned to O–H vibrations absorbed on the surface of Fe nanostructures. In sample A_2,_ four new bands at 1085 and 950, 802, and 466 cm^−1^ have been appeared, which correspond to the asymmetric stretching vibration of Si–O–Si, stretching vibration of the silica shell Si–OH, symmetric stretching vibrations of Si–O–Si, and the Fe–O–Si stretching vibration, respectively. In sample A_3_, the C–Cl bond absorption is expected to be about 800 cm^−1^ which is covered by Si–O–Si symmetric stretching vibration band. The new bands appearing in the 2981 cm^−1^, which is partially covered by the wide range of the O–H band, and the band at 1400 cm^−1^ are attributed to the C(sp^3^)–H stretching, and bending vibrations. In sample A_4_, bands related to the stretching vibrations of the first and second types of amines appear at 3379 and 3440 cm^−1^. This area is also entirely covered with wide O–H band. In sample A_5_, the new band that appeared in 1652 cm^−1^ is related to the absorption of C = O of the amide group.

**Figure 4:**
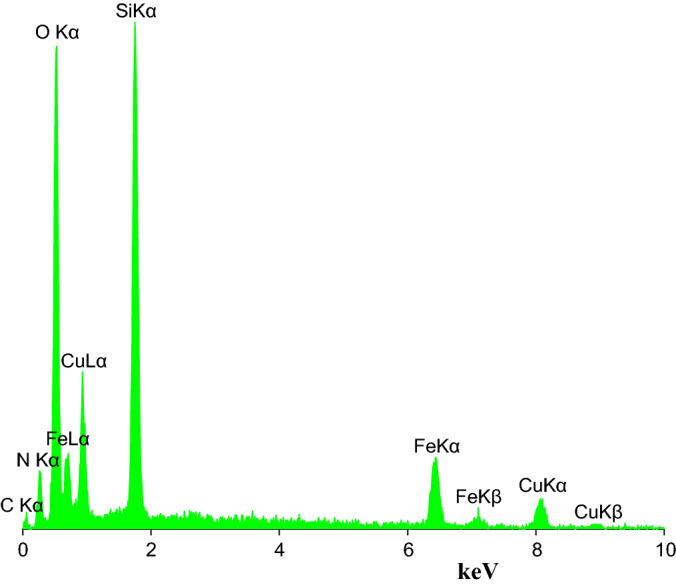
EDS spectrum of Cu(II)MNPs.

Energy-dispersive X-ray spectroscopy (EDS) analysis shows copper ions in the structure of the catalyst, along with C, N, O Si, and Fe atoms (Fig. 4). The quantitative results from EDS analysis of Cu(II)MNPs are given in Table 1.

**Table 1:** Quantitative results from EDS spectrum of Cu(II)MNPs.

Elements	C	N	O	Si	Cl	Fe	Cu	Total
W %	18.21	3.89	60.34	9.59	0.11	4.96	2.89	100.00
A %	25.08	4.60	62.40	5.65	0.05	1.47	0.75	100.00

**Figure 5:**
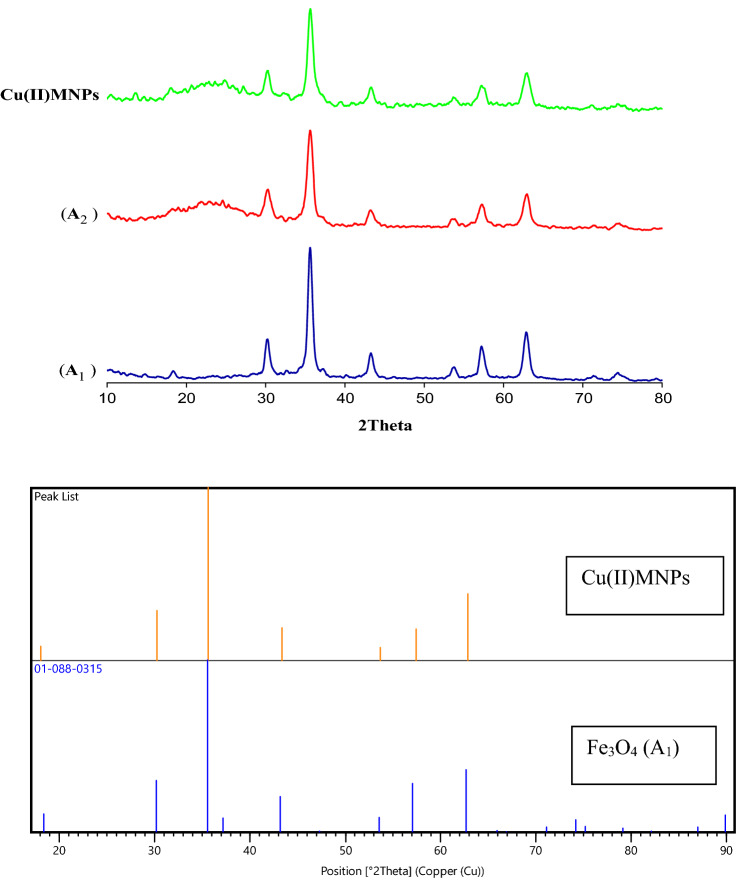
Up: XRD patterns of nanoparticles for Fe_3_O_4_ (A_1_), Fe_3_O_4_@SiO_2_ (A_2_), and Cu(II)MNPs, Comparison of reference Fe_3_O_4_ XRD pattern with Cu(II)MNPs sample.

The XRD patterns of Fe_3_O_4_, Fe_3_O_4_@SiO_2_, and Cu(II)MNPs are shown in Fig. 5. In Fe_3_O_4_ sample, the relative position, and intensities of all the diffraction peaks have been matched with the standard XRD pattern (JCPDS card no.01–088-0315), and the six characteristic diffraction peaks at 30.19°, 35.51°, 43.37°, 53.77°, 57.09°, and 62.76° are observed, which correspond to the (220), (311), (400), (422), (511) and (440) Miller indices, respectively. A broad peak is observed in the range of 20–28, indicating the formation of an amorphous silane shell around Fe_3_O_4_. On the other hand, due to the scattering and small amount of copper ions in the catalyst structure, no new peak is observed in the spectrum of the final sample.

The peak list of the samples A_1_, A_2_ and A_5_ has been given in Table 2.

**Table 2:** XRD Peak list of the samples.

Samples	Pos. [°2Th.]							
Fe_3_O_4_	18/3373	30/1994	35/5115	43/3765	53/7754	57/0974	62/7676	71/2443
Fe_3_O_4_@SiO_2_	30/2538	35/7492	43/1618	53/6972	57/4111	63/0912	74/3352	–
*Cu(II)MNPs*	18/0460	30/2285	35/6139	43/3352	53/7059	57/4608	62/9059	–

This is a fact that magnetite (Fe_3_O_4_), and maghemite (γFe_2_O_3_) present a cubic structure and lattice parameters very close together; for this reason, it is difficult to differentiate these structures even if both phases exhibit high crystallinity. However, some authors report that in the XRD pattern associated with the maghemite phase, there exist two additional peaks located at 23.77° (210) and 26.10° (211). These intensities can be used to differentiate the magnetite phase^25^. We did not observe these two peaks in the XRD obtained (Fig. 5). To prevent the formation of other phases of iron oxide (e.g., γ-Fe_2_O_3_ and FeO) in the co-precipitation method, we removed the oxygen from the reaction environment during the formation of the magnetic nanoparticle by applying a nitrogen atmosphere as suggested in literature^26^.

**Figure 6:**
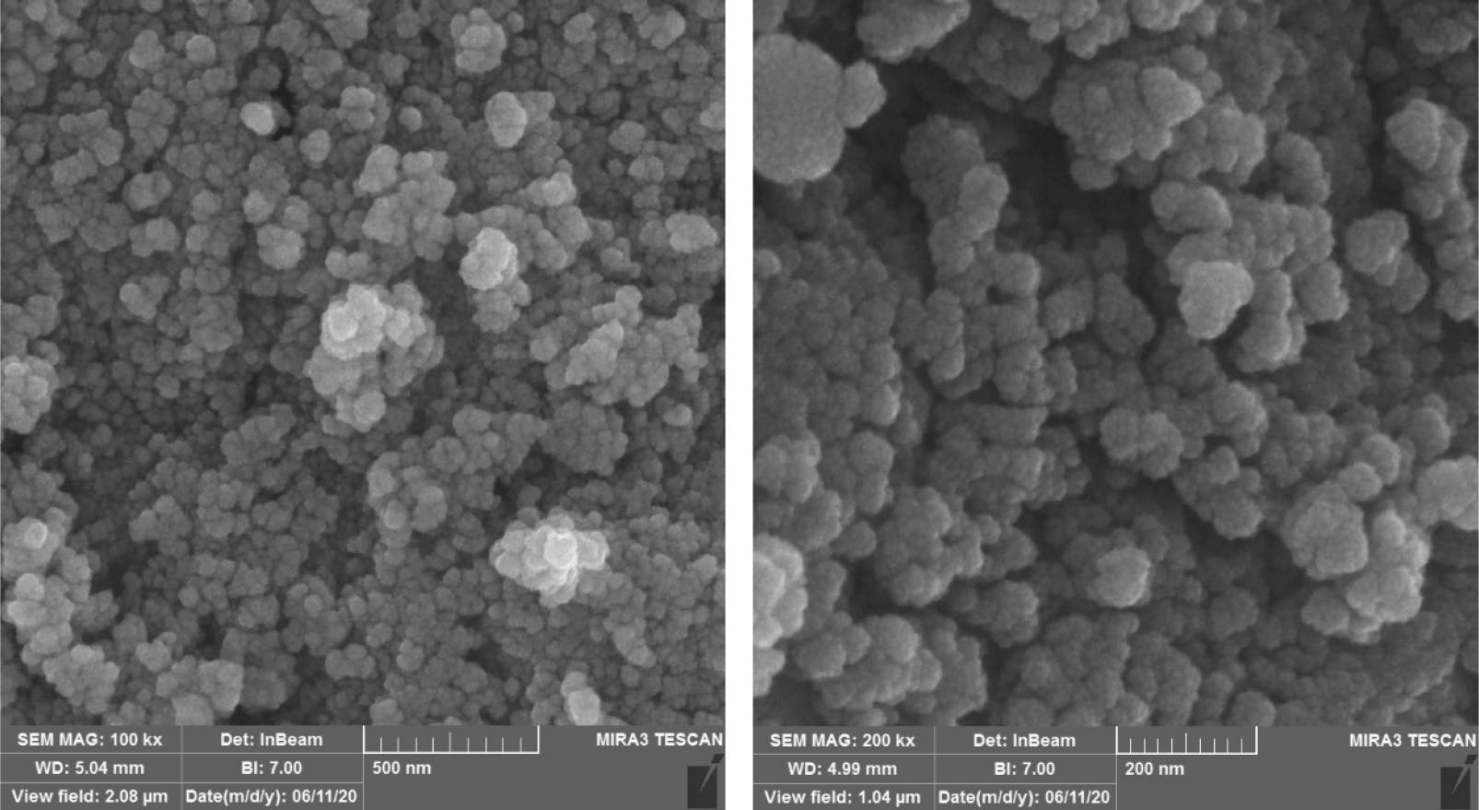
FE-SEM images. Left side: Fe_3_O_4_, right side: Cu(II)MNPs.

Field emission scanning electron microscopy (FE-SEM) images in Fig. 6 show the nanoparticles morphology. According to these images, the diameter of Fe_3_O_4_ particles is approximately 26 ± 8 nm. The diameter is 42 ± 10 nm for Cu(II)MNPs particles. This increase in size is mainly due to the multi-layered coating of silica and functionalization by organic matter around the core.

**Figure 7:**
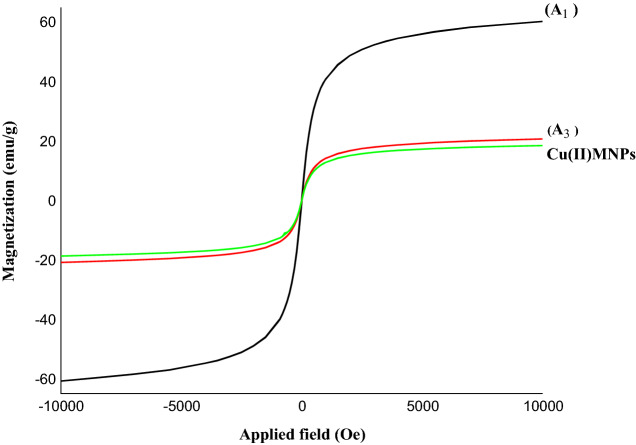
The magnetic hysteresis loops for Fe_3_O_4_ (A_1_), Fe_3_O_4_@SiO_2_–(CH_2_)_3_Cl (A_3_), and Cu(II)MNPs.

Vibrating-sample magnetometry (VSM) has been performed to investigate the magnetic properties of Fe_3_O_4_ NPs (A_1_), Fe_3_O_4_@SiO_2_–(CH_2_)_3_Cl (A_3_), and Cu(II)MNPs with thick silica shell at room temperature. In Fig. 7, the hysteresis loops characteristic of superparamagnetic behavior can be observed for all samples. Super-paramagnetism implies re-dispersion of the magnetic nanoparticles in solution without the occurrence of severe aggregation that ferromagnetic nanoparticles usually suffer from.

**Figure 8:**
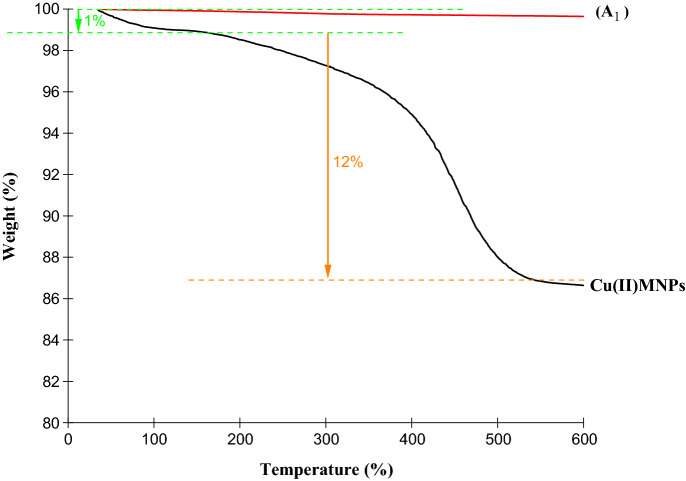
TGA curves for Fe_3_O_4_ (A_1_) and Cu(II)MNPs.

The thermogravimetric analysis (TGA) curves in Fig. 8 provide information on the thermal stability and material weight loss by temperature. In sample Cu(II)MNPs, in the temperature range of 35–170° C, 1% of the mass is reduced due to the loss of tiny amounts of water and solvent molecules that were adsorbed. The second stage begins gradually with the burning of the organic part of the catalyst at 170 °C and ends at 540 °C. A 12% reduction in mass has been observed, which is related to the organic materials stabilized on the catalyst.

We investigated the performance of the catalyst by applying it to the click reaction and forming 5-membered heterocycles, and performed the reaction between an alkyne and an azide in aqueous and organic environments.

**Scheme 2:**
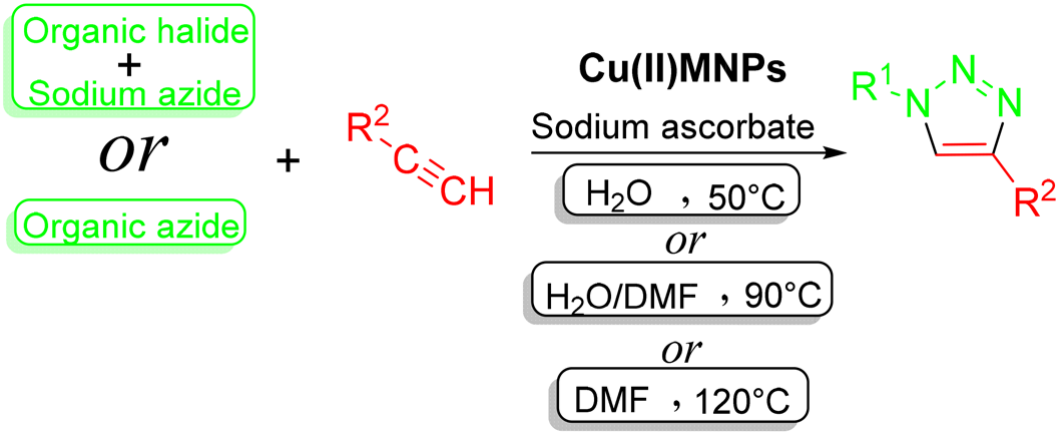
Synthesis of 1,2,3-triazole derivatives.

Some azides are first made separately, and then added to the reaction mixture. If organic halides are used instead of prefabricated azides, the reaction is performed by adding sodium azide under one-pot conditions. Also, the addition of sodium ascorbate is necessary to convert copper(II) to copper(I) (Scheme 2).

**Scheme 3:**
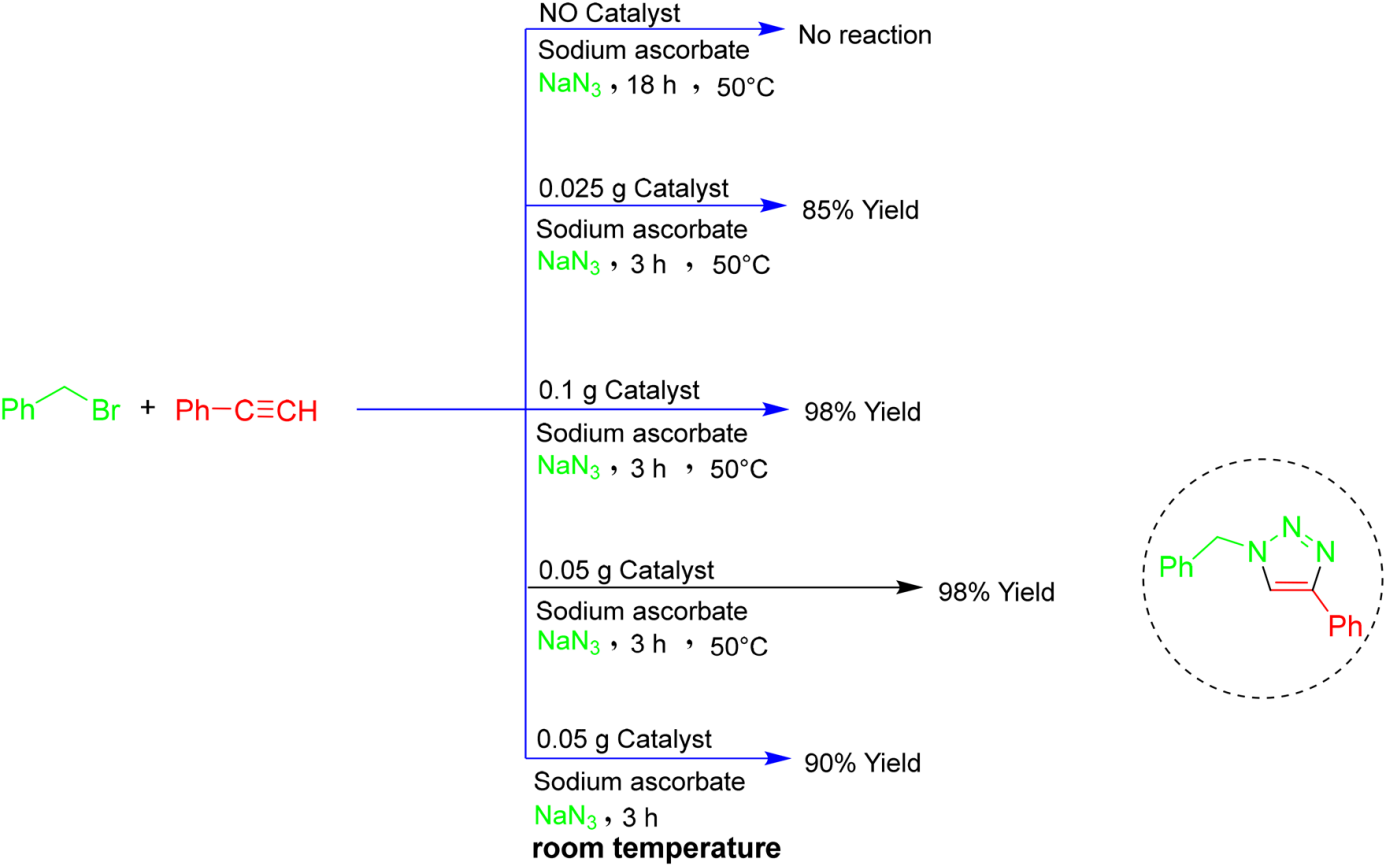
The amount of catalyst used along with the effect of temperature on the reaction efficiency.

In Scheme 3, the amount of catalyst and the effect of temperature on the reaction between phenyl acetylene and benzyl bromide as a model reaction is optimized. The highest yield is obtained in 3 h at 50 °C, by using 0.05 g catalyst or more for one mmole of reactants. We considered this value as the optimal value. Also, in the absence of the catalyst (even by applying a temperature of 50 °C), the reaction did not take place after 18 h. On the other hand, in 3 h and with the optimal amount of the catalyst, lowering the temperature causes the reaction yield to decrease.

The scope of the method has been investigated by using different starting materials (Tables 3, 4). According to the results of Table 3, it is observed that better leaving of bromide group than chloride in organic halides, facilitates the reaction conditions and the products are formed under milder conditions. Also, if an acetylene with less space congestion was used, the reaction proceeded smoothly. The best result in the shortest time was related to the use of benzyl bromide and propargyl alcohol. The results from using pre-formed organic azides has been listed in Table 4.

**Table 3:** Synthesis of 1,2,3-triazole derivatives from organic halides.

Entry	Organic halide	Alkyne	Solvent	Time (h)	Temp (°C)	Triazole	Yield (%)	Melting point (°C)
1			H_2_O	3	50		98	132
2			H_2_O	2	50		98	75–78
3			H_2_O	3	50		98	75–78
4			H_2_O	18	50		Trace	116
			H_2_O/DMF	4	90		90	
5			H_2_O/DMF	4	90		85	116
6			H_2_O	3	50		98	169–171
7			H_2_O	2	50		95	111–113

**Table 4:** Synthesis of 1,2,3-triazole derivatives from organic azides.

Entry	Organic azide	Alkyne	Solvent	Time (h)	Temp (°C)	Triazole	Yield (%)	Melting point (°C)
8			H_2_O	6	50		94	138–139
9			H_2_O	6	50		91	202–203
10			H_2_O	6	50		95	235
11			H_2_O	6	50		88	237
12			H_2_O	4	50		85	231–233
13			H_2_O/DMF	4	90		88	225–227
14			H_2_O	4	50		85	165–167
15			H_2_O	18	50		–	–
			DMF	8	120		94	146

Table 5 shows the comparison between the catalytic activity of Cu(II)MNPs and some other heterogeneous and homogeneous catalytic systems reported before in the reaction of benzyl bromide, NaN_3_, and phenylacetylene. According to Table 5, in addition to improving the efficiency and reaction time in this work, the reaction environment is more green.

**Table 5:** Comparison of catalytic activity of Cu(II)MNPs with other heterogeneous and homogenous copper catalytic systems.

Entry	Catalyst	Catalyst (mol %)	Time (h)	Temp (°C)	Yield (%)	Solvent	References
1	CuSO_4_.5H_2_O	10	2	50	98	H_2_O	This work
2	Cu(OAc)_2_	10	2	50	83	H_2_O	This work
3	CuCl	10	2	50	89^a^	H_2_O	This work
4	CuI	10	2	50	92^a^	H_2_O	This work
5	Cu/C	10	48	r.t	65	dioxane	Bruce et al. (2006)^35^
6	Cu_2_O/C	5	2	r.t	82	i-PrOH/H_2_O	López-Ruiz et al. (2013)^36^
7	Cu-Zeolite	10	15	r.t	83	toluene	Chassaing et al. (2007)^37^
8	Cu-Chitosan	10	6	r.t	90	H_2_O	Anil Kumar et al. (2015)^38^
9	Cu-Alginate	21	18	r.t	98	H_2_O	Rajender Reddy et al. (2007)^39^
10	Cu-Hydroxyappatite	5	16	50	95	H_2_O	Masuyama et al. (2011)^40^
11	Cu(II)MNPs	0.05^b^	3	50	98	H_2_O	This work

^a^Without the addition of sodium ascorbate.

^b^In grams.

**Figure 9:**
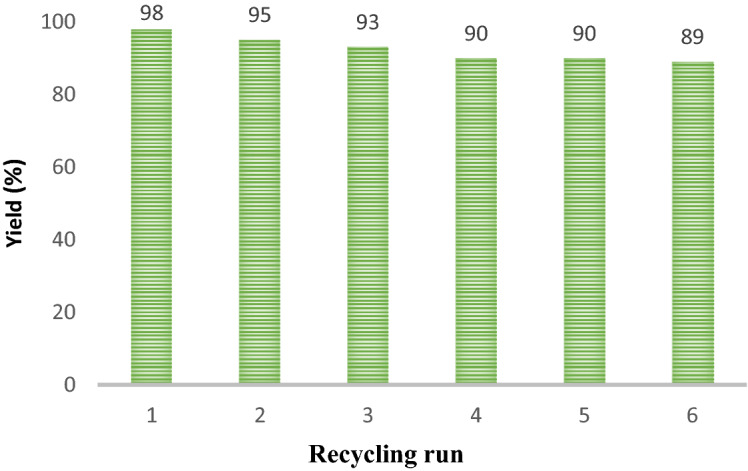
Recycling of the catalyst.

Figure 9 investigates the catalyst reusability in the reaction between phenylacetylene, benzyl bromide, and sodium azide. The reaction time for each step was 3 h. The results showed no significant reduction in efficiency up to at least six stages of recovery and reuse of the catalyst. After each run, the catalyst was separated with an external magnet and washed once or twice with ethyl acetate, ethanol, and distilled water. Then, to dry and reuse, it was placed in an oven at 80 °C for 12 h. At each step, the reaction efficiency was calculated separately, and no significant reduction in reaction efficiency was observed after repeated cycles.

**Scheme 4:**
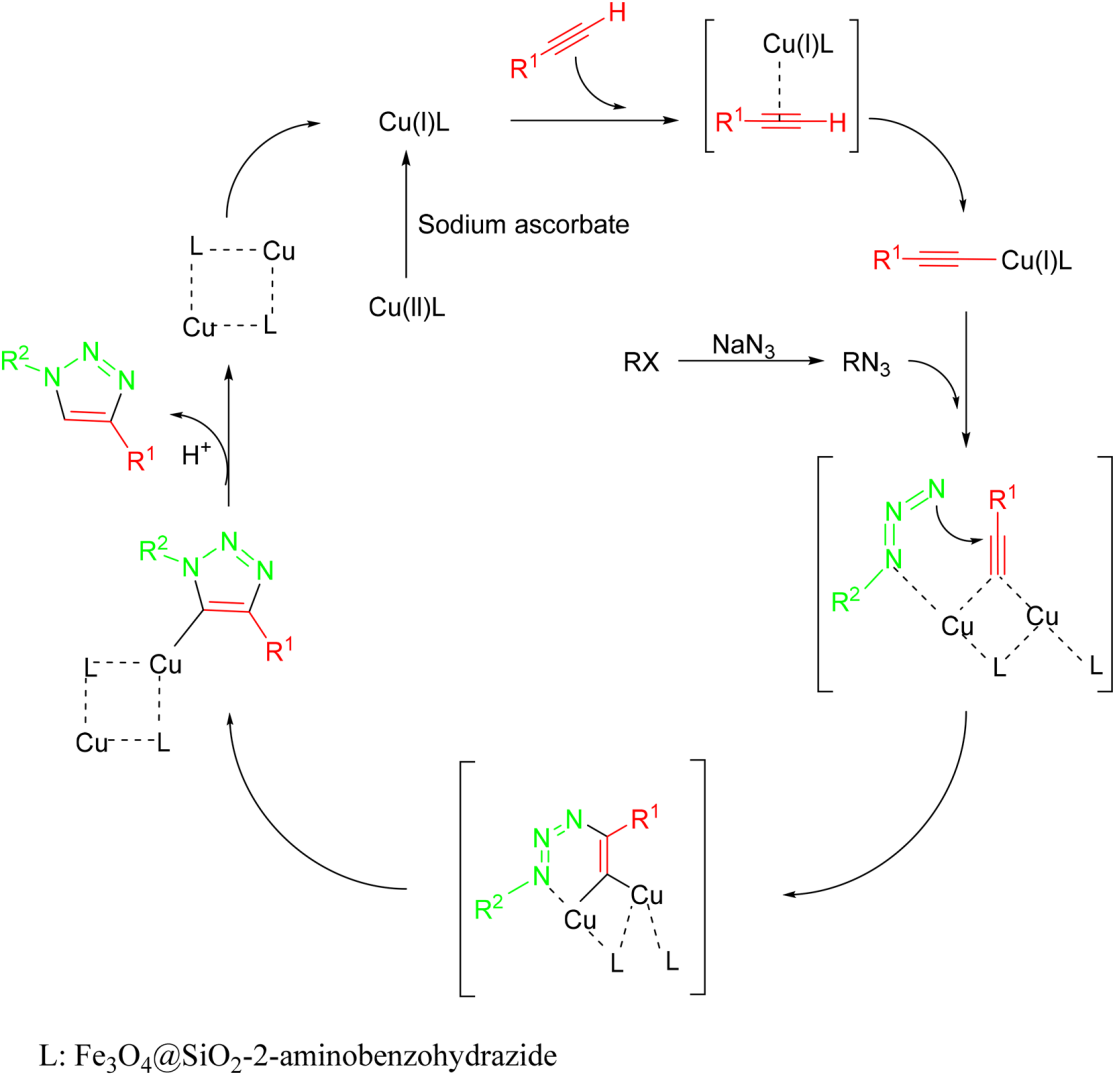
Proposed mechanism for (CuAAC).

A plausible mechanism for the reaction is given in Scheme 4. It is postulated that the copper-catalyzed azide-alkyne cycloaddition (CuAAC) is carried out by the 1,3-dipolar cycloaddition reaction, between alkyl azide and acetylenic compound. The suggested mechanism has been studied and approved elsewhere^27–29^.

The present catalyst is supposed to supply Cu(I) ions in situ from the reaction of Cu(II)MNPs with ascorbic acid. Propyltrimethoxysilane moiety in the catalyst structure was intended to be the boundary between the chelating group and the inorganic substrate. The purpose of this work was preparing a tridentate ligand linked to a heterogenous magnetic substrate (capable of being separated from the reaction mixture easily), binding cupper ions effectively, to avoid a leaching in the solvent.

## Experimental

### General

All materials used were commercially available and purchased from Merck and used without any additional purification. ^1^H NMR spectra were recorded on a Varian-INOVA 500 MHz spectrometer using DMSO as solvent at room temperature. Chemical shifts δ were reported in ppm relative to tetramethylsilane as an internal standard. FTIR spectra of samples were taken by KBr pellets using a Shimadzu FTIR-8400S spectrophotometer. Surface morphology was observed using a Tescan MIRA3 Field Emission Scanning Electron Microscopy (FE-SEM). Thermogravimetric analyses were performed under an air atmosphere in a TGA Q50 with a rate of ca. 20 °C per minute. Magnetization measurements were carried out on a BHV-55 vibration sample magnetometer (VSM). Elemental analysis was performed using EDS analysis, which was recorded by Tescan VEGA/XMU. X‐ray diffraction (XRD) powder patterns were obtained using a Bruker D8 ADVANCE, with Cu Kα radiation. The melting points of the prepared derivatives were measured by an Electrothermal 9100 apparatus reported without any correction.

### Synthesis of Fe_3_O_4_ by co-precipitation method

Fe_3_O_4_ nanoparticles were prepared according to the procedure reported in the literature with some modifications. We synthesized Fe_3_O_4_ nanoparticles by the co-precipitation method. FeCl_3_.6H_2_O (0.02 mol, 5.406 g) and FeCl_2_.4H_2_O (0.01 mol, 1.988 g) were dissolved in 80 mL of deionized water previously heated to 85˚C. then, 25% Ammonia solution (0.32 mol, 12.7 mL) was added to the reaction container dropwise under nitrogen gas and with vigorous stirring. The magnetic nanoparticles were separated from the solution by an external magnet and washed several times with deionized water and ethanol. Finally, the black Fe_3_O_4_ powder was dried for 12 h at 80 °C in a vacuum oven^30,31^.

### Coating with silica shell by Stober method

One g of Fe_3_O_4_, 20 mL of distilled water, and 60 mL of ethanol were added to the reaction container. Then, the reaction mixture was placed in an ultrasonic bath for half an hour. After that, 3 mL of 25% ammonia solution was added to the reaction container and stirred by a mechanical stirrer at room temperature. Simultaneously, 5 mL of tetraethyl orthosilicate was added dropwise to the reaction container. After 12 h, the product was collected by an external magnet and washed several times with distilled water and ethanol, and then dried in an oven at 60°C^32,33^.

### Functionalization of the surface of the silica shell with 3-chloropropyltrimethoxysilane

One g of Fe_3_O_4_@SiO_2_ and 40 mL of ethanol were added to the reaction container and placed in an ultrasonic bath for 10 min. The temperature was adjusted to 70 °C, and 1.75 mL of CPTMS was added dropwise to the reaction mixture. After 24 h, the reaction product was collected by an external magnet and washed several times with ethanol, and then dried in an oven at 60 °C.

### Substitution of hydrazine hydrate with chlorine positions on the nanomagnetic substrate

The product obtained from the previous step was added to 30 mL of ethanol. 0.2 mL of hydrazine hydrate was added dropwise to the reaction mixture at 70 °C and vigorous stirring. After overnight, the product was collected by an external magnet and washed several times with water and alcohol, and then dried in an oven at 60 °C.

### Increase of isatoic anhydride to hydrazine positions

30 mL of ethanol was added to the product obtained from the previous step, and the reaction mixture was stirred continuously with a mechanical stirrer. Then, isatoic anhydride (2.4 mmol, 0.4 g) was added to the reaction container at 70 °C. After one day, the product was separated by an external magnet and washed several times with acetone, and dried in an oven at 60 °C.

### Loading of copper (II) metal cation

The product obtained from the previous step was added to 25 mL of ethanol, and the reaction temperature was set at 70 °C. Then, 0.24 g of copper (II) acetate was added to the reaction container, and the reaction mixture was stirred vigorously. After 24 h, the reaction product was separated by an external magnet and washed several times with water and ethanol. Finally, Cu(II)MNPs were dried at 60 °C.

### Method of preparation of organic azides from aniline derivatives

To a solution of *p*-TsOH·H_2_O (1.62 g, 9 mmol) in H_2_O (9 mL) was added ArNH_2_ (1 mmol). After stirring for 1 min, anhydrous NaNO_2_ (0.621 g, 9 mmol) was added gradually for 5 min. The resulting solution was then stirred for 2–60 min until the starting ArNH_2_ disappeared as monitored by TLC (eluent: benzene–EtOH, 9:1). To the resulting solution, anhydrous NaN_3_ (0.104 g, 1.6 mmol) was added. An immediate emission of N_2_ was observed. Solid aryl azides were filtered, washed with H_2_O (50 mL), and dried. Oily azides were extracted with Ethyl acetate (3 × 10 mL), dried (Na_2_SO_4_), filtered, and the solvent was removed in a rotary evaporator under reduced pressure^34^.

### Copper-catalyzed 1,3-dipolar cycloaddition of azides to alkynes

First, NaN_3_ (1.1 mmol, 72 mg) was added to 1 mmol of organic halides to prepare the organic azides, then 1 mmol of alkyne was added to the corresponding mixture. In the next step, the reaction mixture was added to the pre-prepared suspension of 3 mL of distilled water or DMF, catalyst (0.5 g), and sodium ascorbate (the size of a Scoopula tip). The reaction temperature was adjusted according to the solvent type, and the reaction was followed by TLC. At the end of the reaction, 30 mL of distilled water was added and extracted with ethyl acetate (3 × 10 mL), dried with MgSO_4_, and filtered. Finally, the solvent was evaporated by rotary.

If organic azides are used in the reaction instead of organic halides, NaN_3_ is not added to the reaction mixture.

## Conclusion

Synthesis of 1,2,3-triazole derivatives was performed using a copper(II)-coated magnetic core–shell nanoparticles Fe_3_O_4_@SiO_2_ modified by isatoic anhydride, as a new high-performance catalytic system. Due to its magnetic properties, it is effortless to separate this catalyst by applying an external magnetic field. On the other hand, due to the complete removal of the catalyst from the reaction environment, it is possible to use this catalyst in drug synthesis applications. High thermal stability makes it possible to use this catalyst in organic reactions under high temperatures. Dispersability in organic and aqueous solvents provides conditions for use in both environments. The simple and easy manufacturing method of this catalyst, along with the ability to recover and reuse, makes it economical.

The high efficiency of the click reaction in polar solvents and the high boiling point of the solvent to facilitate the reaction led to the selection of DMF as an alternative solvent for those reactions that do not occur in an aqueous environment. Also, the dispersion of catalyst particles in DMF is higher than in water.
